# Distance to alveolar crestal bone: a critical factor in the success of orthodontic mini-implants

**DOI:** 10.1186/s40510-019-0273-1

**Published:** 2019-05-13

**Authors:** Ramzi Haddad, Maria Saadeh

**Affiliations:** 1 Division of Orthodontics and Dentofacial Orthopedics, American University of Beirut Medical Center, Beirut, Lebanon; 2 Department of Orthodontics, Lebanese University Faculty of Dental Medicine, Beirut, Lebanon; 3 Department of Forensic Odontology, Anthropology and Human Identification, Lebanese University Faculty of Dental Medicine, Beirut, Lebanon

**Keywords:** Orthodontic mini-implants, Alveolar crestal bone, Root proximity

## Abstract

**Background:**

To evaluate the success rate of orthodontic mini-implant (MI) in relation to implant characteristics, mainly implant distance to alveolar crestal bone (AC) and root proximity (RP) to adjacent teeth.

**Methods:**

Two hundred sixty MIs (209 in maxilla, 51 in mandible) were categorized into success (*n* = 229) and failure (*n* = 31) groups. Distances from MI to the most adjacent tooth (DT) and to AC level (DC) were measured on periapical radiographs taken with the orthoradial projection technique. Appropriate statistical tests (chi-square, *t* test, logistic regression) were applied.

**Results:**

DC measurements were statistically significantly greater in the success group (7.46 ± 1.7 mm) compared to 3.43 ± 0.81 mm in the failure group. Root proximity was not associated with miniscrew failure. Patient age, mini-implant site, and DC were significant predictors of mini-implant failure (*p* < 0.001), which decreased significantly with increasing age (Coef = − 0.345; *p* = 0.013) and when the mini-implant was placed between premolars (*p* = 0.028) or between premolar and first molar (*p* = 0.045)*.* The probability of failure also decreased with increasing DC distance (Coef = − 3.595; *p* < 0.001).

**Conclusion:**

The distance to alveolar crest was strongly associated with long-term stability. More apical placement of the MI from the crest would be compatible with a denser and thicker bucco-lingual/palatal bone level.

## Introduction

Temporary anchorage devices (TADs) include miniplates and mini-implants (MIs). The latter are most commonly used because of small size, ease of placement and removal at various sites in the oral cavity, and their acceptance by patients. Nevertheless, compared to endosseous implants, they have a reduced success rate, ranging between 70.7 and 95.2% [1, 2]. MI failure has been linked to factors related to the patient, the screw design, and the placement technique.

Reported patient-related risk factors include younger age [3], high mandibular plane angle [4], mandibular retrusion [5], and most importantly the site of implant placement [3, 6]. Consistently, greater failure rates have been observed in the mandible compared to the maxilla [3, 7–10] Within the maxilla, failure prevalence in more posterior sites is likely associated with reduced cortical bone thickness [3], which was found significantly higher with successful MIs (1.34 ± 0.35 mm) compared to failed implants (0.99 ± 0.09 mm) [11].

Design-related factors have been investigated extensively. Lower success rates with smaller diameter and shorter MI length (1–1.1 vs 1.5–2.3-mm diameter; 6-mm vs 8-mm length) [7, 10] presumably relate to decreased surface area and implant to bone contact. However, a meta-analysis [3] and additional studies [12, 13] disclosed no significant effect of implant thread diameter or length [3], although shorter miniscrews have shown higher failure rates.

Technique-related factors include method of placement, root proximity, and MI loading. Maximal insertion torque of 5 to 10 Ncm was deemed optimal for MI success, greater amounts reportedly causing stress, necrosis, and local ischemia [3, 11, 14]. Current clinical evidence suggests similar success rates of self-tapping and self-drilling miniscrews [15]. In addition, immediate and delayed loading as well as healing periods did not significantly affect MI stability [3, 7, 16]. Also, lower success rate of secondary insertion (44.2%) was reported in comparison to primary insertion (80.4%) [10].

The orientation of placement at 90° to the bone surface has been advocated as the most stable and resistant to failure [17], but a recent study revealed higher primary stability at 45° when the miniscrews were loaded by shear force, and at 90° when pullout force was applied [18]. The soft tissue at the site of placement has also been cited as impacting implant stability. Most available studies advocate insertion in attached gingival tissue over soft mucosal tissue to avoid irritation or inflammation [9, 19, 20], but others did not disclose a significant difference when MIs were placed in the mandibular buccal shelves [21].

MI success rate has been linked to operator experience [6] and surgical techniques, which are associated with a steep learning curve to maintain optimal placement procedures [12]. Higher failure rates have been connected with placement on the right side of the mouth [8], possibly because of the prevalence of right-handed patients and the associated easier site access for better hygiene on the left [8].

An increasingly reported predictor of MI failure is root proximity, a factor also related to the operator’s experience and judgment of proper site in sufficient inter-radicular bone. The definition of “root proximity” denotes root contact, whereby the MI apex or body overlays radiographically the lamina dura, but is not in actual contact with the root [2, 22]. While root contact reportedly yielded three times more failure than no contact [3], the association with root proximity remains unclear, especially since conflicting results have been published in studies using cone-beam computed tomography (CBCT) to assess MI success [23, 24].

We observed in clinical practice more failure when MIs were placed too close to the alveolar crest (AC), a heretofore not clarified issue. We hypothesized that the proximity to AC was an additional factor affecting the stability of orthodontic MIs. Therefore, the aim of this study was to evaluate the relationship between MI success rate and its proximity to AC, as well as the association between success and other factors including gender, age, jaw, side and site of placement, and MI type.

## Materials and methods

This retrospective study was approved by the Institutional Review Board ((ID#: OTO.RH.01). From a total of 293 MIs placed in 260 patients, right and left implants had been inserted in 33 patients. A separate comparison of the parameters analyzed in the study was conducted between right and left sides in these patients and revealed non-significant statistical differences. Accordingly, one of the MIs on either side was randomly selected for inclusion in the final sample of 260 MIs.

More implants were positioned in the maxilla (*n* = 209) than in the mandible (*n* = 51) in 131 males and 129 females (mean age, 23.45 years; range, 13–51.4 years). Two MI types were used: type 1, AbsoAnchor (Dentos, Daegu, Korea)—diameter 1.4 mm, length 8 mm; type 2, Imtec (3 M, USA)–diameter 1.8 mm, length 8 mm.

All MIs were inserted under local anesthesia by one orthodontist (RH) without mucoperiosteal incision or flap, at the level of the attached gingival line, using a manual self-drilling method. The insertion angulation was at 30–35° to the horizontal. Based on clinical judgment in the individual situations, 4 placement sites were selected: (1) between canines and first premolars (C-Pm1), (2) between first and second premolars (Pm1–Pm2), (3) between second premolars and permanent first molars (Pm2-M1), and (4) between first and second permanent molars (M1–M2). Periapical radiographs were taken before and after MI placement with the orthoradial-projection technique using an X-ray holder (Rinn, Dentsply, USA) and a digital radiographic machine (Instrumentarium Dental Company, Tuusula, Finland). After confirming initial stability, the MIs were immediately loaded with a power chain (around 150 g). The implantation was considered successful when the MI remained stable throughout force application, until completion of the required orthodontic movement. Failure was recorded at the time of observation in the mouth.

### Radiographic evaluation

**Fig. 1:**
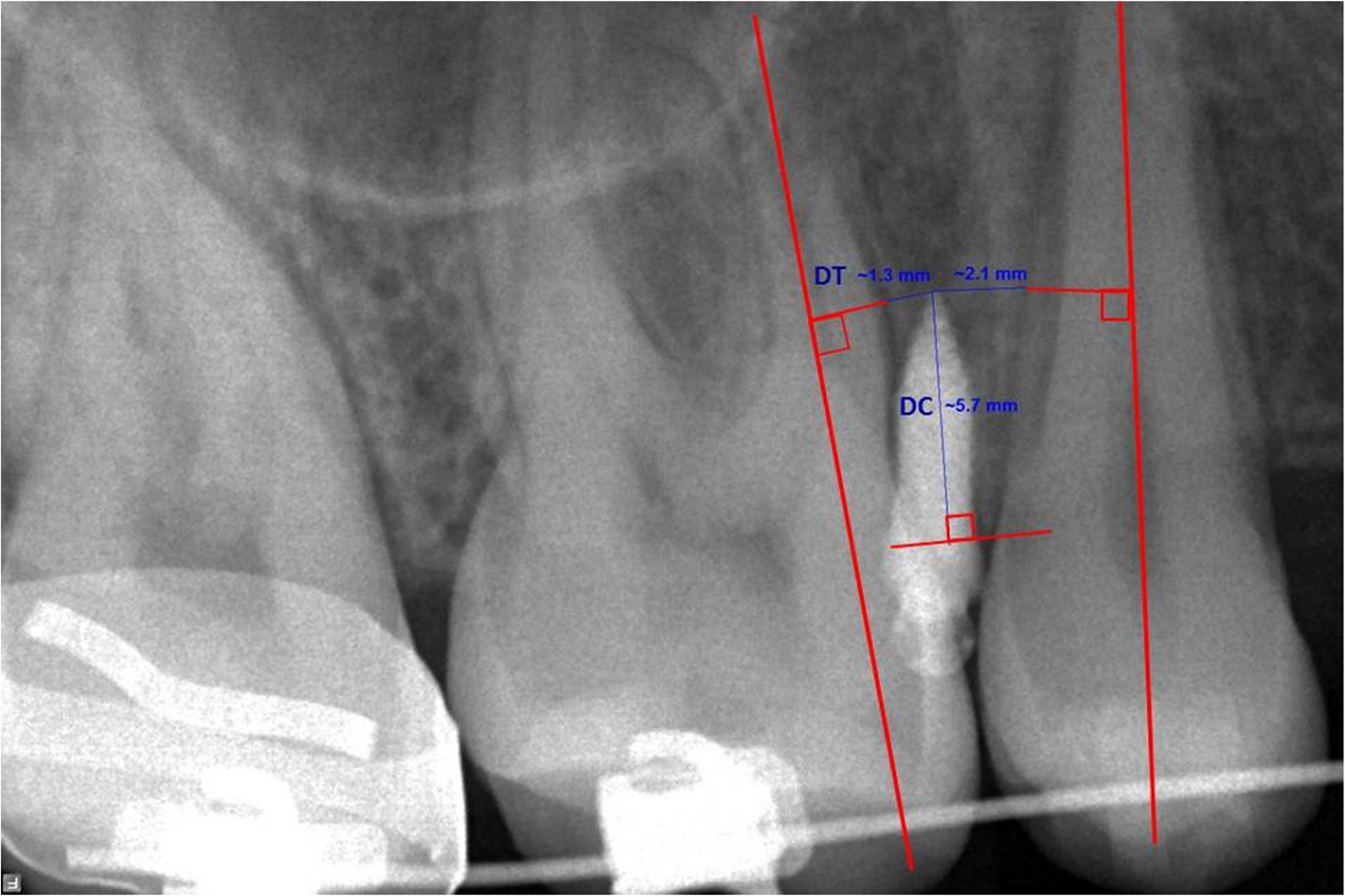
Radiographic evaluation using a periapical radiograph

The radiographs were processed using the manufacturer’s program (Cliniview Software, Version 9.3.0.6); their ratios to actual size were 1:1. The following measurements were recorded (Fig.1):

- Perpendicular distance from the MI tip to the root of the most adjacent tooth (DT); the perpendicular was projected to the long axis of the adjacent root.
- Perpendicular distance from the MI tip to the alveolar crestal bone level (DC); the perpendicular was projected to the tangent to the alveolar crestal tip. The shortest distance to either the mesial or distal root was considered in the statistical computations.

The radiographic measurements were performed blindly, without knowledge of MI failure. To evaluate the error of magnification, we measured the height and width of the premolar bracket on 20 randomly selected radiographs, as well as the length of 20 MIs on another 20 randomly selected radiographs. The brackets and MIs were compared with the actual standard height (3.0 mm) and width (3.1 mm) of the bracket and the length of the MI (8 mm for both types). The average magnification for bracket height and width were 0.015 + 0.06 mm and 0.01 + 0.06 mm, respectively. The magnification for the MI was 0.1 ± 0.09 mm. Accordingly, the direct measurements on radiographs were adopted for statistical computations.

To assess intra-examiner reliability, the measurements were repeated by the same investigator at a 2-week interval on a randomly selected subsample of 44 periapical films (20% of total sample).

### Statistical analysis

A test of normality revealed that the data were normally distributed in both groups. The chi-square test was used to analyze the relationship between success rate and categorical variables including gender (male, female), age (≤ 20; 20–30; ≥ 30 years), jaw (maxilla, mandible), side (right, left), site (C–Pm1; Pm1–Pm2; Pm2–M1; M1–M2) and MI type (type 1, type 2). The *t* test served to study the difference in DC and DT between the two outcome groups (success and failure). Multiple logistic regression modeling was used to assess the predictors of failure. Intra-examiner reliability in measuring DT and DC was assessed using the two-way mixed effects intraclass correlation coefficient for absolute agreement. The level of significance was set at 0.05. All statistical analyses were conducted using IBM® SPSS® v. 23.0 statistical package.

## Results

The intraclass correlation coefficients between the two readings were 0.972 for DT and 0.964 for DC, indicating high intra-examiner reliability.

The overall success rate was 88.1% (31 failed MIs out of 260). No statistically significant differences were found between success groups across the different categories, including gender, age, jaw, side, site, and MI type (Table 1).

**Table 1:** Descriptive statistics and comparison of success rates between different categories

Factor		*N*	Success rate (%)	Chi-square	*p*
Gender	Male	131	91.7	3.641	0.056
	Female	129	84.1		
Age (years)	< 20	112	87.5	1.493	0.494
	20–30	103	86.4		
	> 30	45	93.6		
Jaw	Maxilla	209	90	3.568	0.088
	Mandible	51	80.4		
Side	Right	143	88.2	0.163	0.705
	Left	117	87.6		
Site	C–Pm1	25	88.0	3.853	0.278
	Pm1–Pm2	30	86.7		
	Pm2–M1	131	91.6		
	M1–M2	74	82.4		
MI type	Type 1	113	85	1.854	0.173
	Type 2	147	90.5		

*Significant, *p* < 0.05

*C* canine; *Pm1*, *Pm2* first, second premolars; *M1*, *M2* first, second molars

Type 1, AbsoAnchor; type 2, Imtec

DT was not statistically significantly different between the success and the failure group (1.69 ± 1.01 mm vs. 1.40 ± 0.56 respectively, *p* = 0.018), in the pooled sample, as well as between jaws and MI type (*p* > 0.05, Table 2). DC differed significantly between the 2 outcome groups (*p* < 0.001): measurements were statistically significantly greater in the success group (7.46 ± 1.7 mm), the average measurements being nearly twice greater than in the failure group (3.43 ± 0.81 mm) **(**Table 3**)**. This pattern was also observed within each of the jaws separately and with either type of mini-implants for DC.

**Table 2:** Comparison of DT distance (mm) between the two groups in the pooled sample, in each jaw separately and by mini-implant type

			Jaw				Type			
	Pooled		Maxilla		Mandible		1		2	
	Success	Failure	Success	Failure	Success	Failure	Success	Failure	Success	Failure
*N*	229	31	188	21	41	10	133	14	96	17
Min	0.3	0.5	0.3	0.5	0.7	0.8	0.3	0.8	0.4	0.5
Max	3.6	2.6	3.6	2.6	3.6	2.5	3.6	2.6	3.3	2.3
Mean	1.6	1.42	1.60	1.44	1.62	1.39	1.73	1.52	1.42	1.34
SD	0.77	0.56	0.75	0.55	0.87	0.59	0.78	0.55	0.71	0.56
*t*	1.621		0.966		0.798		0.986		0.458	
p	0.112		0.335		0.429		0.326		0.648	

Type 1, AbsoAnchor; type 2, Imtec

*Significant, *p* < 0.05; **significant, *p* < 0.01

**Table 3:** Comparison of DC distance (mm) between the two groups in the pooled sample, in each jaw separately and by mini-implant type

			Jaw				Type			
	Pooled		Maxilla		Mandible		1		2	
	Success	Failure	Success	Failure	Success	Failure	Success	Failure	Success	Failure
*N*	229	31	188	21	41	10	133	14	96	17
Min	2.1	2.1	3.3	2.1	2.1	2.2	2.7	2.2	2.1	2.1
Max	10.9	5.0	10.9	5.0	10.2	4.4	10.9	4.7	10.4	5.0
Mean	7.46	3.43	7.7	3.37	6.26	3.58	7.81	3.20	6.96	3.63
SD	1.7	0.81	1.67	0.9	1.37	0.59	1.61	0.96	1.71	0.63
*t*	21.819		18.712		6.144		15.728		14.401	
*p*	< 0.001**		< 0.001**		< 0.001**		< 0.001**		< 0.001**	

Type 1, AbsoAnchor; type 2, Imtec

*Significant, *p* < 0.05; **significant, *p* < 0.01

In the logistic regression predicting failure, patient age, mini-implant site, and DC were significant predictors of mini-implant failure while controlling for the effects of gender, type, jaw, side, and DT (*p* < 0.001; Table 4). The probability of failure decreased significantly with increasing age (Coef = − 0.345; *p* = 0.013), when the mini-implant was placed between premolars or between premolar and first molar (*p* = 0.028 and 0.045, respectively), and with increasing DC distance (Coef = − 3.595; *p* < 0.001).

**Table 4:** Multivariate logistic analysis showing associations between mini-implant success and explanatory variables (*n* = 260)

Associated variables	Coef.	Std. err.	95% CI	*p* value
	Failure (yes/no) ǂ			
Constant	25.684	7.927	[10.148; 41.220]	0.001**
Age	− 0.345	0.139	[− 0.618; − 0.073]	0.013*
Gender (*male)*				
Female	1.841	1.302	[− 0.710; 4.393]	0.157
Site *(canine–premolar 1)*				
Premolar 1–premolar 2	− 6.180	2.804	[− 11.675; − 0.674]	0.028*
Premolar 2–molar 1	− 4.919	2.453	[− 9.728; − 0.110]	0.045*
Molar 1–molar 2	− 1.715	2.421	[− 6.461; 3.030]	0.479
Type (*Imtek)*				
AbsoAnchor	− 2.303	1.493	[− 5.228; 0.623]	0.123
Jaw (*maxilla*)				
Mandible	− 0.008	1.303	[− 2.563; 2.546]	0.995
Side (*right*)				
Left	0.427	0.974	[− 1.481; 2.335]	0.661
DT	0.558	0.867	[− 1.141; 2.258]	0.519
DC	− 3.595	0.855	[− 5.270; − 1.919]	< 0.001**
Likelihood ratio *χ*^2^	146.45			
Degrees of freedom	10			
Prob > *F*	< 0.001**			
Pseudo *R*^2^	0.8074			

Age recorded in years; DT and DC recorded in mm

*(Base)* refers to the base outcome all other categories are compared to

*Coef.* regression coefficient, *Std. err.* standard error, *DT* distance to adjacent root, *DC* distance to alveolar crest level

^*^Statistically significant at *p* < 0.05; ^**^statistically significant at *p* < 0.01.

## Discussion

The main contribution of this study was the finding that the success rate of the MIs was higher with a greater distance between the implant and the alveolar crest, applying to both jaws and to both types of MIs. The rate of success (88.1%) corresponded to the mid-range of the success rates reported in other studies (70.7% to 95.2%) [1, 2] and is close to the 87.8% weighted mean survival rate of maxillary MIs related in a meta-analysis [3].

The clinical implication would be to insert the screw away from the crestal edge at a level where a thicker layer of bone would account for the observed stability. Combining this directive with the indication to position the MI within the attached keratinized gingiva for stability and long-term maintenance without inflammation [9, 19, 20], an optimal apical angulation of the MIs (30–35° to the horizontal) would be warranted. Although such an angulation has been proposed to avoid root damage during placement [25, 26], our findings suggest that it would also be essential to maximize MI stability. Further research focused on this aspect is indicated.

Root proximity has been widely associated with MI failure, more in the mandible than in the maxilla [22, 27]. In 2 prior studies using CBCT technology, MI success was also associated with a greater distance from root surface [24, 27]. In our study the distance (DT) from MI to root surface did not differ between success and failure groups irrespective of jaw or MI type (Table 2). The discrepancy with our findings may relate to the available space and operator-sensitive method as the MI insertion is usually within a limited inter-radicular space and is planned to allow leeway for movement of a tooth towards its adjacent, such as the placement of the MI closest to the mesial surface of the first molar prior to its distalization.

Despite the higher success rate in males than females (91.7% and 84.1%, respectively), the lack of statistical significance suggests that gender is not a factor in the failure of MIs, supporting prior conclusions [3, 7, 8, 25]. As corroborated in most studies [3, 5, 7, 25], patient age also did not impact the MI success rate, although this rate increased with age (from 87.5% under age 20 years to 93.3% over age 30 years). Yet, when controlling for other variables, age emerged as a predictor of implant failure in the logistic regression analysis, joining the conclusion of Yao et al. who ascribed greater risk of failure to MIs placed in patients younger than 35 years, using a generalized estimating equation [25].

Success rates were not statistically significantly different between the maxilla and the mandible (90% and 80.4% respectively), in agreement with previous findings [4, 6, 15, 26], but also conflicting with systematic reviews suggesting greater failure rates in the mandible compared to the maxilla [3, 7]. The conclusions may have been affected by the disproportion in sample sizes in favor of greater success in the maxilla [7].

Our findings of no statistically significant difference in MI success between right (88.8%) and left (87.2%) sides are in concordance with the most recent systematic review [3] and do not concur with reports of better success on either the left [4, 8] or the right side [13]. The results relating a higher success rate for MIs placed between the second premolar and the first molar (91.6%) and a lower success rate between the two molars (82.4%) may be associated with different bone density existing between the second premolar and first molar compared to that between the two molars, along with the possibility of better hygiene more anteriorly than posteriorly. While the bivariate analysis showed placement between the premolars comparable to other sites, the multivariate analysis depicted the interpremolar and premolar-molar sites as predictors of success.

The investigated MIs had the same length (8 mm), but their diameters differed. The wider MI (1.8 mm, Imtec®) showed greater success rate (90.5%) compared to the other (1.4 mm, AbsoAnchor®; 85%), but the difference was not statistically significant. Published reports are contradictory. One meta-analysis suggested no significant effect of implant thread diameter on failure rate [3]; yet another meta-analysis indicated that MIs of smaller diameter (1–1.1 vs 1.5–2.3 mm) had significantly lower success rates [7]. Research should be focused on whether co-variates rather than diameter alone impact the success rate.

The outcomes are specific to the conditions applied in the present population, whereby the MI was inserted at about 35^o^. However, should the implant be at a different angle, the tip and neck would be at different distances from the alveolar crest, possibly influencing the success rate of the MI.

Measurements on periapical two-dimensional images may be affected by potential projection errors, as variations in the mesio-distal and vertical directions of the X-ray beam may modify DT and DC, respectively. To counter such errors, we used standardized methods with properly positioned X-ray holders to best approximate the axes of the teeth to real anatomy. Imaging would be best with 3-D technology; however, the caveats about increased radiation with CBCT records precluded consideration of this tool by the Institutional Review Board. However, the high intracorrelation coefficients regarding measurement reproducibility on properly taken periapicals reflect the adequacy of these radiographs, which are universally used in similar studies. Also, consideration of the magnification effect disclosed minimal differences in the magnification of brackets and MIs, close 1:1 ratio of measurements that were made to a single point, the tip of the MI, not a line or surface.

The retrospective nature of this study imposed several inevitable limitations, including the inability to control for various factors known to affect MI stability such as insertion torque, patient oral hygiene, local gingival inflammation, and smoking [25, 28]. The possible over-representation of MIs with an acceptable distance from adjacent roots may be indirectly related to the discrepancy in sample size between the success (*n* = 229) and failure (*n* = 31) groups. Lower failure representation reflects clinical realities, related to increasing adherence to proper MI placement. Yet, validation of our findings through future research should generate a solid base for the above-inferred clinical recommendations.

## Conclusions

1. Implant stability is associated with the distance from the MI to the alveolar crestal bone.
2. Along with distance to alveolar crest, age and MI site were significant predictors of failure.
3. Root proximity was not associated with the failure of MIs as suggested by previous studies.
4. The clinical corollary to placing the MI within the attached gingiva but away from the alveolar crest would be to angulate the MI apically to position it in a thicker bucco-lingual/palatal level of bone.

## Abbreviations

AC: Alveolar Crest
C: Canine
CBCT: Cone-beam computed tomography
DC: Perpendicular distance from the MI tip to the alveolar crestal bone level
DT: Perpendicular distance from the MI tip to the root of the most adjacent tooth
M1: First molar
M2: Second molar
MI: Mini-implant
Pm1: First premolar
Pm2: Second premolar
TAD: Temporary anchorage device
